# Amyloid beta is associated with carotid wall echolucency and atherosclerotic plaque composition

**DOI:** 10.1038/s41598-024-64906-8

**Published:** 2024-06-28

**Authors:** Dimitrios Delialis, Georgios Georgiopoulos, Simon Tual-Chalot, Lasthenis Angelidakis, Evmorfia Aivalioti, Georgios Mavraganis, Kateryna Sopova, Antonios Argyris, Peggy Kostakou, Christina Konstantaki, Maria Papaioannou, Diamantis Tsilimigras, Konstantinos Chatoupis, Achilleas A. Zacharoulis, George Galyfos, Fragiska Sigala, Konstantinos Stellos, Kimon Stamatelopoulos

**Affiliations:** 1 Department of Clinical Therapeutics, Alexandra Hospital, Medical School, National and Kapodistrian University of Athens, PO Box 11528, 80 Vas. Sofias Str., Athens, Greece; 2 Biosciences Institute, Vascular Biology and Medicine Theme, Faculty of Medical Sciences, Newcastle University, Newcastle Upon Tyne, UK; 3 Department of Cardiovascular Research, European Center for Angioscience (ECAS), Heidelberg University, Ludolf-Krehl-Straße 13-17, D-68167 Mannheim, Germany; 4 Department of Cardiology, University Hospital Mannheim, Mannheim, Germany; 5 First Department of Propaedeutic Surgery, Hippocrateion Hospital, Medical School, University of Athens, Athens, Greece; 6 Second Cardiology Department, Evangelismos Hospital, Athens, Greece; 7 German Centre for Cardiovascular Research (DZHK), Partner Site Heidelberg/Mannheim, Mannheim, Germany; 8 Department of Cardiology, Angiology, Haemostaseology and Medical Intensive Care, University Medical Centre Mannheim, Medical Faculty Mannheim, Heidelberg University, Mannheim, Germany

**Keywords:** Cardiology, Risk factors, Biomarkers

## Abstract

Circulating amyloid-beta 1–40 (Αb40) has pro-atherogenic properties and could serve as a biomarker in atherosclerotic cardiovascular disease (ASCVD). However, the association of Ab40 levels with morphological characteristics reflecting atherosclerotic plaque echolucency and composition is not available. Carotid atherosclerosis was assessed in consecutively recruited individuals without ASCVD (*n* = 342) by ultrasonography. The primary endpoint was grey scale median (GSM) of intima-media complex (IMC) and plaques, analysed using dedicated software. Vascular markers were assessed at two time-points (median follow-up 35.5 months). In *n* = 56 patients undergoing carotid endarterectomy, histological plaque features were analysed. Plasma Αb40 levels were measured at baseline. Ab40 was associated with lower IMC GSM and plaque GSM and higher plaque area at baseline after multivariable adjustment. Increased Ab40 levels were also longitudinally associated with decreasing or persistently low IMC and plaque GSM after multivariable adjustment (*p* < 0.05). In the histological analysis, Ab40 levels were associated with lower incidence of calcified plaques and plaques without high-risk features. Ab40 levels are associated with ultrasonographic and histological markers of carotid wall composition both in the non-stenotic arterial wall and in severely stenotic plaques. These findings support experimental evidence linking Ab40 with plaque vulnerability, possibly mediating its established association with major adverse cardiovascular events.

## Introduction

Despite the progress in atherosclerotic cardiovascular disease (ASCVD) risk assessment and the emergence of new treatment strategies, ASCVD remains the primary cause of morbidity and mortality in the Western world^1^. This underscores the existence of residual risk partly mediated by unedified, yet pro-atherosclerotic pathways not targeted by current standard care therapies^2^. An increasing body of evidence indicates that amyloid-beta 1–40 (Αb40) peptide is involved in a wide spectrum of pro-atherosclerotic pathways, supporting its clinical relevance as a possible prognostic and therapeutic biomarker in ASCVD^3^. Ab40 is derived from proteolytic cleavage of the amyloid precursor protein (APP) by β-secretase^3^ and exerts pro-inflammatory and pro-atherosclerotic properties^3,4^. We previously showed that circulating Ab40 is associated with arterial stiffening in young adults^5^, atherosclerosis in both the general population and patients with coronary artery disease (CAD)^5,6^, and the progression of subclinical carotid atherosclerosis in postmenopausal women^7^. Notably, Ab40 levels provide incremental prognostic and reclassification value beyond traditional risk factors and risk scores, for mortality and major adverse cardiovascular events in patients with stable CAD and with non-ST-segment elevation acute coronary syndrome (NSTE-ACS)^8^. These clinical data support experimental evidence indicating that Ab40 is involved in the continuum of ASCVD and vascular ageing and suggests a clinical utility of this peptide in ASCVD. Given that the vulnerable plaque is defined as the atherosclerotic phenotype prone to rupture, the ultimate step mediating the majority of ASCVD events^9^, exploring associations of Ab40 blood levels with plaque characteristics comprising or reflecting components of this vulnerable phenotype is clinically relevant and may provide mechanistic new insights to the link between Ab40 and increased ASCVD events^3,5,10^. However, despite clinical evidence consistently linking Ab40 plasma levels with adverse ASCVD events^5,10,11^, its association with markers of plaque vulnerability is missing. Carotid plaque echolucency reflects a more lipid-rich atherosclerotic plaque and intraplaque hemorrhage^12,13^ and is considered as a marker of plaque vulnerability, while increased echogenicity may indicate advanced calcification linked with less symptomatic plaques^14–17^. Plaque echolucency identified with the quantitative gray-scale median (GSM) is associated with increased risk of future stroke^18^ and ASCVD events^19^ and could be used to guide intervention in asymptomatic carotid artery stenosis^20,21^. Similarly, intima-media complex GSM is gaining increasing interest due to its prognostic value for future adverse events and its wider applicability irrespective of the presence of plaque^22,23^. To provide mechanistic proof of concept of Ab40 as a marker of plaque composition, we investigated the associations of plasma levels of Ab40 with markers of plaque echolucency and their progression in patients without established ASCVD and plaque calcification in samples from endarterectomy.

## Results

### Population characteristics

In our study, we enrolled 342 participants (*n* = 140 males, 40.9%), with a mean age of 57.7 (12.6) years, with a prevalence of 37.1% (*n* = 127) for hypertension, 48% (*n* = 164) for hyperlipidemia, 30.4% (*n* = 104) for smoking and 12.2% (*n* = 42) for diabetes mellitus. Regarding carotid ultrasound data, at least one carotid plaque was found in 39.7% of the population and intima-media complex (IMC) in all the participants. Plaque GSM values ranged from 1.2 to 88, IMC GSM values from 1.0 to 89.0, maximal plaque area from 3.16 to 88.9 mm^2^ and total plaque area from 3.16 to 318.5 mm^2^. Subjects with Ab40 levels in the highest tertile had marginally higher prevalence of male sex (*p* = 0.062) and higher presence of carotid plaque atherosclerosis, higher total and maximal area plaque and lower glomerular filtration rate (GFR), HDL-C and plaque GSM (*p* < 0.05 for all) (Table 1).

**Table 1:** Descriptive characteristics of the population (*n* = 342) by Ab40 tertiles (i.e., lower vs. highest).

	Total population (*n* = 342)	Lower (*n* = 228)	Highest (*n* = 114)	*p*-value
Sex [male], *n* (%)	140 (40.9)	85 (37.3)	55 (39.3)	0.062
Age [years], mean (SD)	57 (12.6)	56.9 (12.8)	59.3 (12.0)	0.095
Smoking, *n* (%)	104 (30.4)	71(31.7)	33 (30.3)	0.751
Hypertension, *n* (%)	127 (37.1)	78 (34.7)	49 (44.5)	0.178
Hyperlipidemia, *n* (%)	164 (47.9)	107(47.6)	57 (50)	0.651
Hypolipidemic treatment, *n* (%)	94 (27.4)	60 (26.7)	34 (30.9)	0.574
Diabetes mellitus, *n* (%)	42 (12.2)	23 (10.2)	19 (17.3)	0.15
BMI [kg/m^2^], mean (SD)	27.5 (4.7)	27.4 (4.7)	27.5 (4.7)	0.95
GFR [ml/min/m^2^], mean (SD)	108.3 (43.1)	117.3 (42.4)	105.8 (45.6)	0.04
Total cholesterol [mg/dl], mean (SD)	204 (46)	205 (43)	203 (51)	0.649
Triglycerides [mg/dl], median (IQR)	95 (70)	110 (59)	118 (73)	0.268
LDL-C [mg/dl], mean (SD)	125 (40)	130 (39)	130 (43)	0.916
HDL-C [mg/dl], mean (SD)	56 (16)	52 (16)	44 (14)	0.018
hs-CRP [mg/dl], median (IQR)	1.1 (2.07)	1.05 (2.08)	1.2 (2.46)	0.486
Amyloid-beta 1–40 [pg/ml], median (IQR)	49.0 (35.6)	40.95 (24.9–51.6)	91.3 (71.53–118.4)	< 0.001
SBP [mmHg], mean (SD)	125.6 (19.2)	127.1 (18.4)	131.4 (20.7)	0.053
DBP [mmHg], mean (SD)	72.3 (10.8)	72.5 (11.0)	73.3 (10.5)	0.551
Presence of Carotid plaque, *n* (%)	136 (39.7)	82 (37.4)	54 (51.4)	0.022
Plaque GSM, [ median (IQR)	43 (20)	46 (26.3)	38.0 (22.0)	0.037
Maximum plaque area, [mm^2^], median (IQR)	24.8 (26.1)	21.8 (19.0)	28.4 (32.8)	0.017
Total plaque area, [mm^2^], median (IQR)	34.4 (43.9)	30.5 (34.3)	51.2 (53.1)	0.016

*P*-values are derived from independent Student’s *t*-test for continuous variables and the Pearson’s chi squared test for categorical variables. BMI: Body mass index, hs-CRP: high-sensitivity C-reactive protein, GFR: Glomerular filtration rate, GSM: gray-scale median, LDL-C: low-density lipoprotein cholesterol, HDL-C: high-density lipoprotein cholesterol, SBP: Systolic blood pressure, DBP: Diastolic blood pressure.

### Cross-sectional analysis. Ab40 plasma levels are associated with plaque echolucency and extent of carotid atherosclerosis

**Figure 1:**
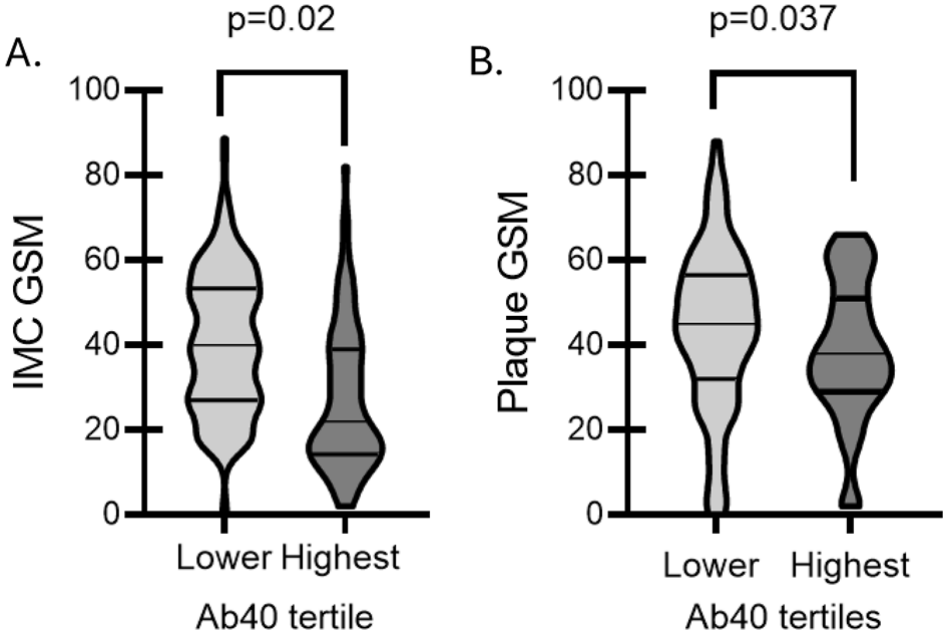
Violin plots of differences in IMC and plaque GSM by Ab40 tertiles. *P*-values are derived from independent samples Mann–Whitney comparing GSM values between highest vs. lower and middle tertile of Ab40 levels. Ab40: Amyloid-beta 1–40, GSM: grey scale median, IMC: intima-media complex.

The linear correlation between Ab40 levels and plaque and IMC GSM was marginally significant in the whole population (Supplementary Fig. 1A, C). Given that GFR is a major determinant of both vascular calcification^24,25^ and Ab40 clearance^3^, we explored this association in the subgroup of patients with GFR ≥ 60 ml/min/m^2^ and found a significantly linear correlation between both IMC and plaque GSM (Supplementary Fig. 1B, D). Increased Ab40 levels were associated with higher odds for lower IMC GSM and lower plaque GSM in all carotid sites (OR = 2.54 increase for highest vs lower Ab40 tertiles 95% CI: 1.35, 4.79, *p* = 0.004 for IMC GSM and OR = 4.78 increase for highest vs lower Ab40 tertiles 95% CI: 1.69, 13.52, *p* = 0.025 for plaque GSM) after adjustment for the core model and sum of carotid wall thickness (Table 2, Fig. 1). Moreover, increased Ab40 levels were associated with increased odds for higher total and maximal plaque area (OR = 3.81 increase for highest vs lower Ab40 tertiles 95% CI: 1.35, 10.75, *P* = 0.011, OR = 4.68 increase for highest vs lower Ab40 tertiles 95% CI: 1.68, 14.21, *P* = 0.002 for maximal and total plaque area, respectively) (Table 2). These associations did not change after further adjustment for hs-CRP and lipid-lowering treatment (Table 2). The interaction between sex and Ab40 levels did not have a significant effect on the carotid markers (*p* > 0.05 for all, Supplementary Table 1).

**Table 2:** Association of Ab40 levels with carotid atherosclerosis indices (*n* = 342).

	Model 1	Model 2
OR (95% CI)		
Maximal plaque area (highest tertile)	3.81 (1.35 / 10.75) *P* = 0.011	4.01 (1.34 / 12.02) *P* = 0.013
Total plaque area (highest tertile)	4.68 (1.68 / 14.21) *P* = 0.002	5.69 (1.77 / 18.22) *P* = 0.003
IMC GSM * (lowest tertile)	2.54 (1.35 / 4.79) *P* = 0.004	4.40 (1.49 / 13.02) *P* = 0.007
Plaque GSM* (lowest tertile)	4.78 (1.69 / 13.52) *P* = 0.025	7.20 (2.08 / 24.92) *P* = 0.002

OR represents the odds ratio for a patient with Ab40 levels in the highest tertile to have carotid atherosclerotic indices (total and max plaque area) in the highest tertile and GSM in the lowest compared to their counterparts with low Ab40 levels (i.e., lower tertiles).

Multivariable model 1 includes sex, age, smoking status, history of hypertension, dyslipidemia, history of diabetes mellitus, and glomerular filtration rate. Model 2: Model 1 plus high-sensitivity C-reactive protein levels and lipid-lowering treatment.

*Multivariable model for IMC and plaque GSM also includes sum of carotid wall thickness.

Abbreviations: hs-CRP: high-sensitivity C-reactive protein, GSM: gray scale median, IMC: intima-media complex.

### Prospective analysis. Ab40 plasma levels are associated with changes in plaque echolucency and the extent of carotid atherosclerosis

**Figure 2:**
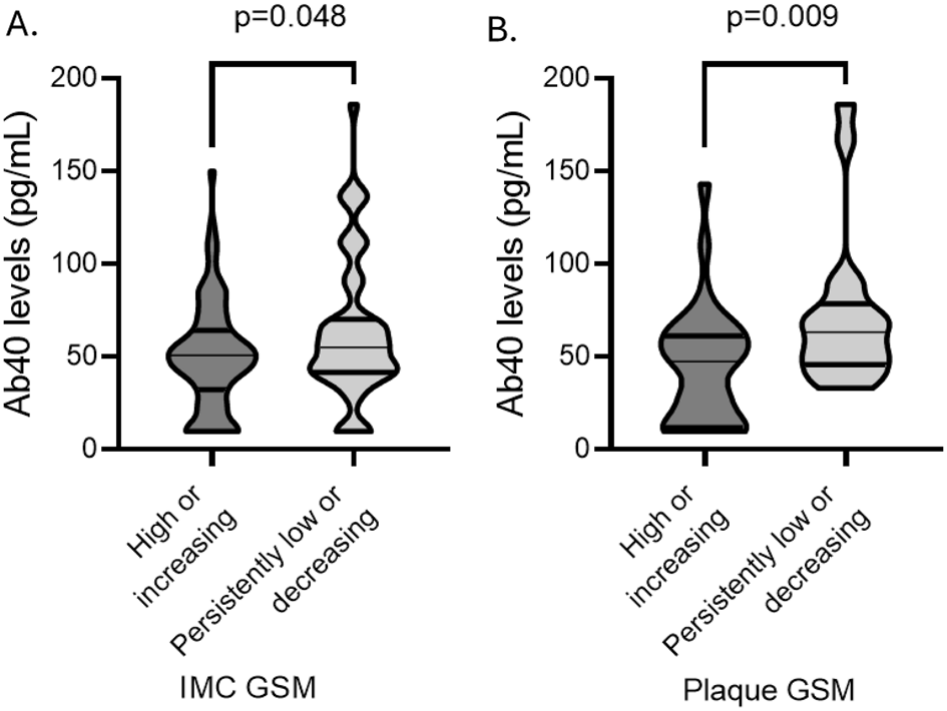
Violin plots of Ab40 levels by Low / decreasing GSM vs. high / increasing A. IMC GSM and B. plaque GSM (i.e., lowest vs higher tertiles). *P*-values are derived from independent samples Mann–Whitney comparing GSM values between highest vs. lower and middle tertile of Ab4040 levels. Ab40: Amyloid-beta 1–40, GSM: grey scale median, IMC: intima-media complex.

After a median follow-up of 35.5 months, the carotid ultrasonography measurements were repeated using the same protocol in a subgroup of these patients (*n* = 148). Patients with available follow-up measurements for the study did not differ from those with only baseline measurements available (Supplementary Table 2). The maximum plaque area and the total plaque area both significantly increased in *n* = 148 patients, whereas the IMC and plaque GSM did not significantly change (Supplementary Table 3). However, in *n* = 70 and *n* = 23 patients, IMC GSM and plaque GSM also decreased, respectively (Supplementary Table 3). Ab40 levels were correlated with lower IMC GSM and with plaque GSM at follow-up (Supplementary Fig. 2A,B) and changes in plaque GSM in persistently low / decreasing GSM group (Supplementary Fig. 2C). Changes in IMC GSM in both groups and plaque GSM in persistently high / increasing GSM group were not correlated with Ab40 levels (Supplementary Fig. 2 D,F). Importantly, increased Ab40 levels were associated with decreasing or persistently low GSM levels of IMC and plaque (OR = 2.78 increase for highest vs lower Ab40 tertiles 95% CI: 1.46, 3.68, *p* = 0.002 for IMC GSM and OR = 4.5 increase for highest vs lower Ab40 tertiles 95% CI: 1.63, 12.44, *p* = 0.004, for plaque GSM) (Table 3, Fig. 2) at follow-up after adjustment for the core model and sum of carotid wall thickness. Moreover, increased Ab40 levels were associated with higher odds for increased or persistently high carotid atherosclerosis burden (OR = 4.49 95% increase for highest vs lower Ab40 tertiles CI: 1.54, 13.09, *p* = 0.006 for delta of maximum plaque area and OR = 5.15 increase for highest vs lower Ab4040 tertiles increase 95% CI: 1.70, 15.58, *p* = 0.004 for delta of total of plaque area) at follow-up after adjustment for the core model (Table 3). These associations did not materially change after further adjustment for hs-CRP and lipid-lowering treatment (Table 3), and the interaction between sex and Ab40 levels did not have a significant effect on the carotid markers (*p* > 0.05 for all, Supplementary Table 4).

**Table 3:** Association of Ab40 levels with the progression of carotid atherosclerosis indices (*n* = 148).

Carotid atherosclerosis	Model 1	Model 2
Pattern of increasing or persistently highest tertile of	OR (95% CI)	
Maximal plaque area	4.49 (1.54 / 13.09) *P* = 0.006	4.80 (1.54 / 15.00) *P* = 0.007
Total plaque area	5.15 (1.70 / 15.58) *P* = 0.004	6.39 (1.85 / 22.14) *P* = 0.003
Pattern of decreasing or persistently lowest tertile of	OR (95% CI)	
IMC GSM min*	2.78 (1.46 / 3.68) *P* = 0.002	6.08 (1.99 / 19.43) *P* = 0.002
PLQ GSM min*	4.50 (1.63 / 12.44) *P* = 0.004	7.20 (2.08 / 24.92) *P* = 0.002

OR represents the odds ratio for a patient with Ab40 levels in the highest tertile to have increasing or persistently high carotid atherosclerotic indices (total and max plaque area) and decreasing or persistently low GSM (IMC and plaque) compared to their counterparts with low Ab40 levels (i.e., lower tertiles).

Multivariable model 1 includes sex, age, smoking status, history of hypertension, dyslipidemia, history of diabetes mellitus, and glomerular filtration rate. Model 2: Model 1 plus high-sensitivity C-reactive protein levels and lipid-lowering treatment.

*Multivariable model for IMC and plaque GSM also includes sum of carotid wall thickness.

Abbreviations: hs-CRP: high-sensitivity C-reactive protein, GSM: gray scale median, IMC: intima-media complex.

### Histological analysis. Ab40 levels are associated with less calcified and less plaques without high-risk features

**Figure 3:**
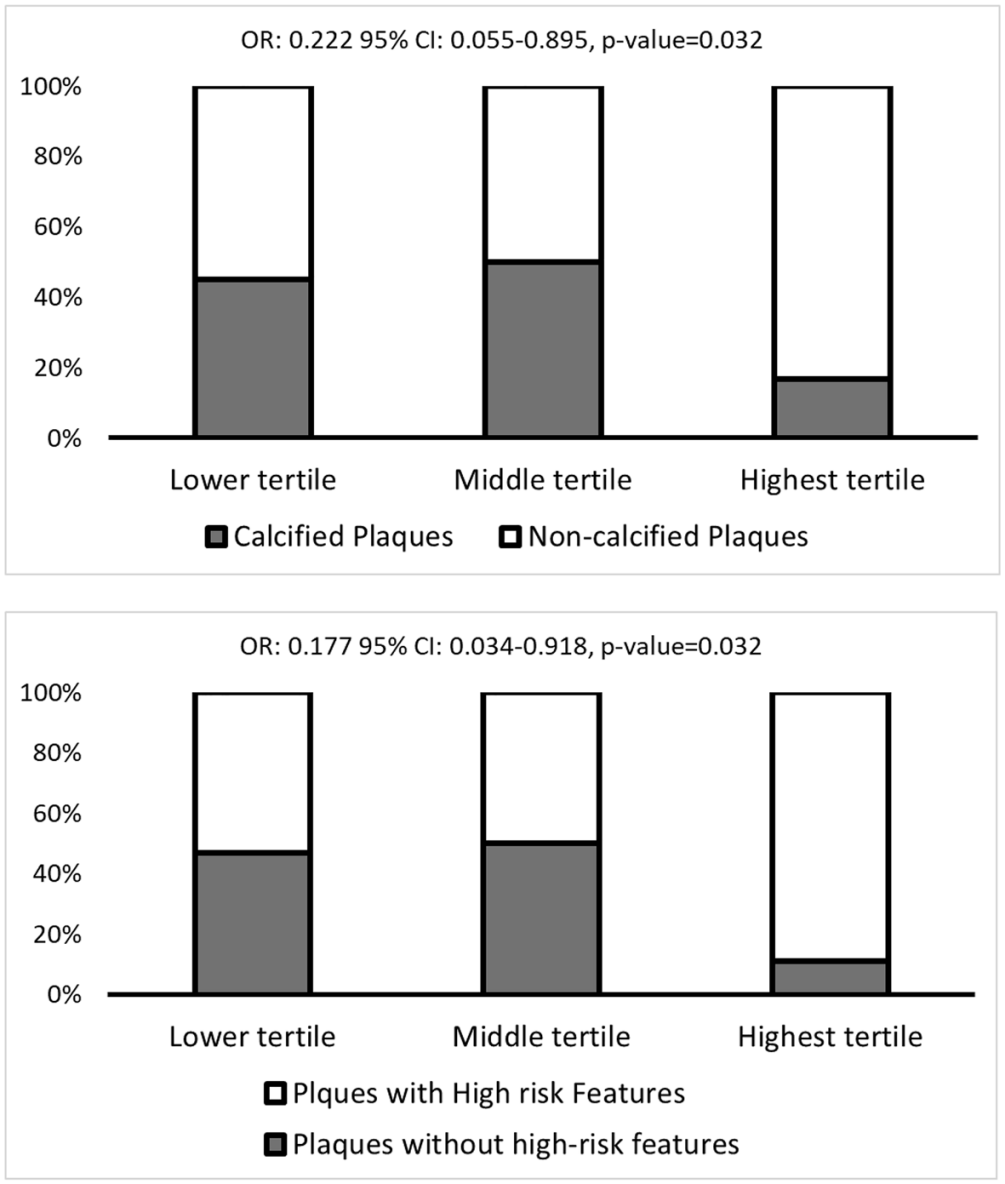
Percentage of patients with A. calcified plaques and B. Plaques without high-risk features plaques by Ab40 tertiles. *P*-values are derived from logistic regression analysis (highest vs lower Ab40 tertile).

Patients with Ab40 levels at the highest tertile had higher incidence of GFR < 60 ml/min/m^2^ without any other differences in demographic or clinical data (Table 4). Regarding plaque characterisation, patients with Ab40 in the highest tertile had lower incidence of calcified plaques (16.7% in the highest Ab40 tertile vs. 47.8% in the lower tertiles, *p* = 0.039, OR = 0.222 95% CI: 0.055–0.895, *p* = 0.034, Figure 3A) and lower incidence of plaques without high-risk features (11.1% in the highest tertile vs 41.4% in lower tertiles, *p* = 0.047, OR = 0.177, 95% CI: 0.034, 0.918, *p* = 0.039, Figure 3B).

**Table 4:** Descriptive characteristics of the population with endarterectomy (*n* = 56) by Ab40 tertiles (i.e., lower vs. highest).

	Lower (*n* = 38)	Highest (*n* = 18)	*p*-value
Sex [male], *n* (%)	17 (44.4)	6 (33.3)	0.563
Age [years], mean (SD)	74.4 (8.6)	76.5 (10.3)	0.445
Smoking, *n* (%)	10 (26.7)	2 (11.1)	0.403
Hypertension, *n* (%)	37 (97.4)	17 (94.4)	0.544
Hyperlipidemia, *n* (%)	8 (21.1)	4 (22.2)	1.0
GFR < 60 ml/min/m^2^, *n* (%)	5 (13.2)	7 (38.9)	0.04
Diabetes mellitus, *n* (%)	10 (26.3)	7 (17.9)	0.366
Coronary artery disease, *n* (%)	19 (50)	9 (50)	1.0
PAD, *n* (%)	7 (18.4)	2 (11.1)	0.413
Statins, *n* (%)	31 (83.8)	14 (87.5)	1.0
ACEi, *n* (%)	27 (71.2)	13 (72.2)	0.385
Antiplatelet, *n* (%)	34 (89.5)	16 (88.8)	0.9
Amyloid-beta 1–40 [pg/ml], median (IQR)	42 (33.4–53.3)	98.3 (81.9–131.4)	< 0.001
Symptomatic carotid stenosis, *n* (%)	22 (57.9)	12 (39.3)	0.573
Angiographic carotid stenosis ≥ 90%, *n* (%)	25 (67.6)	11 (61.1)	0.764
Plaque histopathology status			
Calcified plaques, *n* (%)	18 (47.8)	13 (16.7)	0.039
Hemorrhage, *n* (%)	2 (5.3)	2 (11.1)	0.587
Ulceration, *n* (%)	15 (39.5)	10 (55.6)	0.388
Thrombus, *n* (%)	6 (15.8)	5 (27.8)	0.305
Plaques without high-risk features, *n* (%)	16 (41.4)	2 (11.1)	0.047

*P*-values are derived from independent Student’s *t*-test for continuous variables and the Pearson’s chi squared test for categorical variables. BMI: Body mass index, GFR: Glomerular filtration rate, SBP: Systolic blood pressure, DBP: Diastolic blood pressure. Symptomatic carotid stenosis includes: presence of stroke, brain infarcts, transient ischemic attacks and amaurosis fugax. Carotid plaques were assigned according to the presence of histologic components related to plaque vulnerability i.e., intraplaque hemorrhage, ulcer, or thrombus or with plaque stability i.e. calcification. Plaques without high-risk features were defined as calcified plaques without any unstable features.

## Discussion

In the current study, we report for the first time in humans, that Ab40 plasma levels were associated a. with plaque echolucency, an ultrasonographic marker of plaque instability, in patients without clinically overt ASCVD and b. with histological features of lower plaque calcification and lower incidence of plaques without high-risk features in severely stenotic plaques from patients undergoing endarterectomy. Importantly, the ultrasonographic associations persisted over time for a median follow-up of 35.5 months. Finally, we confirmed that Ab40 levels were associated with carotid atherosclerosis progression not only in postmenopausal women, as previously reported^7^, but also in a wider population of patients with risk factors and without established ASCVD.

Our findings show that increased Ab40 levels are associated with echolucent carotid wall. Although the clinical value of intima-media thickness is still debatable^26,27^, carotid plaque assessment is well established as an associate of incident ASCVD events and is recognised as a risk enhancer of ASCVD by ESC Guidelines, allowing reclassification of patients and more intensive treatment over traditional ASCVD risk factors^26,27^. Beyond the previously reported associations of Ab40 with the presence, extent and progression of carotid atherosclerosis that were also evident in the current study^5–7^, we further report significant data regarding the association of Ab40 with markers of carotid wall (IMC and plaque) composition. Echolucency of carotid plaques reflects a more lipid-rich plaque and is considered as a marker of plaque instability^12,13^, while high echogenicity may reflect increased calcification^14,15^. GSM is the most accurate method to assess echolucency with reproducibility among variable populations and across time frames^28–30^ and has been proposed to guide decisions on invasive treatment of obstructive carotid lesions^20,31^. Carotid IMC and plaque echolucency are inter-related^32^, and both have been associated with an adverse cardiovascular profile and increased ASCVD risk^19,30^, irrespective of the extent of carotid atherosclerosis^33^ and degree of lumen stenosis^34^. In recent studies, IMC GSM is recognised as an additional marker of carotid wall echolucency, associated with atherosclerosis and vascular ageing and being readily measurable in all participants regardless of the presence of carotid plaque^22,23^. Therefore, our findings in patients without established ASCVD, provide novel clinical mechanistic evidence on the pro-atherosclerotic effect of Ab40 and would support the clinical value of this biomarker in a primary prevention population in need for accurate risk stratification.

Moreover, we present the first longitudinal evidence showing that increased baseline Ab40 is consistently associated with higher echolucency of carotid arterial wall over time (35.5 months median follow-up). In agreement with previous evidence, on average, GSM values did not change in our population^35,36^. However, stable values in a population may be driven by opposing directions of GSM changes in specific subgroups. Indeed, in our subgroup with persistently low or decreasing carotid wall GSM, GSM values tended to decrease, while this GSM pattern of change has been suggested to be an adverse feature related with higher incidence of ASCVD events and increased risk of cognitive dysfunction^35,36^. Another point of interest, is our finding that Ab40 levels were associated with carotid atherosclerosis progression in a wider population of patients with risk factors and without established ASCVD than that of postmenopausal women previously reported^7^, expanding the generalizability of the observed association of this peptide with atherosclerosis in humans. Collectively, these results support a consistent association of plasma Ab40 levels with carotid wall echolucency over time and increase the generalizability of clinical evidence linking Ab40 with atherosclerosis in humans. On the other hand, further research in a larger prospective cohort is needed to explore and validate the association of Ab40 with long-term progression towards a high-risk atherosclerotic plaque profile.

In the current study, we provide the first human histological evidence that Ab40 circulating levels are associated with carotid plaque composition and specifically with less calcified plaques, a feature related to less symptomatic plaques^16,17^, and less plaques without high-risk features. Given that increased echogenicity is mainly driven by calcification^14,15^, the results from the ultrasonographic and histologic parts of our study are aligned, supporting the hypothesis that increased plasma Ab40 levels may reflect more vulnerable arterial wall composition ranging from subclinical early atherosclerotic intima-media complex lesions to severe symptomatic plaques. Another common finding observed in both cohorts was the graded rather than linear association between markers of arterial wall composition and Ab40 levels. This may be attributed to the intrinsic properties of amyloid peptides, which exhibit higher toxicity when Ab40 monomers accumulate, forming oligomers and inducing cell injury beyond a concentration threshold of oligomer^37,38^. In support, similar stepwise associations have been previously reported between Ab40 and ASCVD endpoints^5,10,11^. Mechanistically, previous experimental evidence indicates that Ab40 is implicated in the formation of advanced and vulnerable atherosclerotic plaques through platelet phagocytosis, neovascularisation, monocyte stimulation and accelerated foam cell formation^3,4,39^. These functions may mediate clinical results associating Ab40 blood levels with major adverse cardiovascular events independently of traditional risk factors, established risk scores and systemic inflammation as assessed by hs-CRP in both patients with stable CAD and NSTE-ACS^5,10^, supporting its additive clinical utility as a prognostic biomarker in ASCVD. Given the systematic nature of atherosclerosis^26,27^, our findings support this mechanistic link connecting Ab40 with atherosclerotic plaque vulnerability and adverse cardiovascular events, suggesting that Ab40 is implicated in atherosclerotic plaque composition beyond inflammatory burden and other well-established determinants of plaque echolucency^24,25,40,41^. Therefore, these findings mechanistically support the investigation of the prognostic role of Ab40 in the general population and merit further investigation since our population size and relatively low ASCVD risk did not permit event analysis. Finally, these findings could trigger further research assessing Ab40 as a potential therapeutic target. Lifestyle modifications and several cardiovascular drugs, including statins, angiotensin-renin axis inhibitors, b-blockers, and hemodialysis have shown potential of modulating Ab40 levels and metabolism^3^. The effect of changes in Ab40 levels on plaque composition in response to such interventions merits further investigation.

Some limitations should be acknowledged. Firstly, although the sample size of our study is limited, similar sample sizes have been used in previous studies investigating the association of biomarkers with plaque echogenicity^42–45^. Secondly, the prospective results were available in a subgroup of our cohort. However, the descriptive characteristics between the two subgroups with and without follow-up did not differ (Supplementary Table 1). Furthermore, the design of this study being a non-interventional observational study does not permit inference of causal relationships. Additionally, despite multivariate adjustment, residual confounding may affect interpretation of these findings. Moreover, the association between IMC GSM and Ab40 levels seems less potent than plaque GSM. This is not unexpected given that IMT reflects much earlier phenotypically profound stages of atherosclerosis, which probably weakens the potential of Ab40 to capture pathophysiology at this level. Although inflammatory parameters were not available in the histological evaluation of excised plaques, in our population without severe carotid stenosis, we found that the association of Ab40 with GSM remained significant even after adjustment hs-CRP levels, lipid-lowering treatment, GFR and carotid atherosclerosis burden.

In conclusion, our findings provide ultrasonographic and histological evidence indicating that Ab40 levels are associated with markers of carotid wall composition in both the non-stenotic carotid arterial wall and in severely stenotic plaques. Further, our longitudinal data support these findings by indicating consistent associations over time of Ab40 levels with plaque echolucency in patients without clinically overt ASCVD. Collectively, our findings suggest a link between systemic Ab40 levels and plaque vulnerability in humans, which supports previously published epidemiological evidence on its prognostic value for adverse ASCVD events.

## Methods

### Population

#### Athens Cardiometabolic study

**Figure 4:**
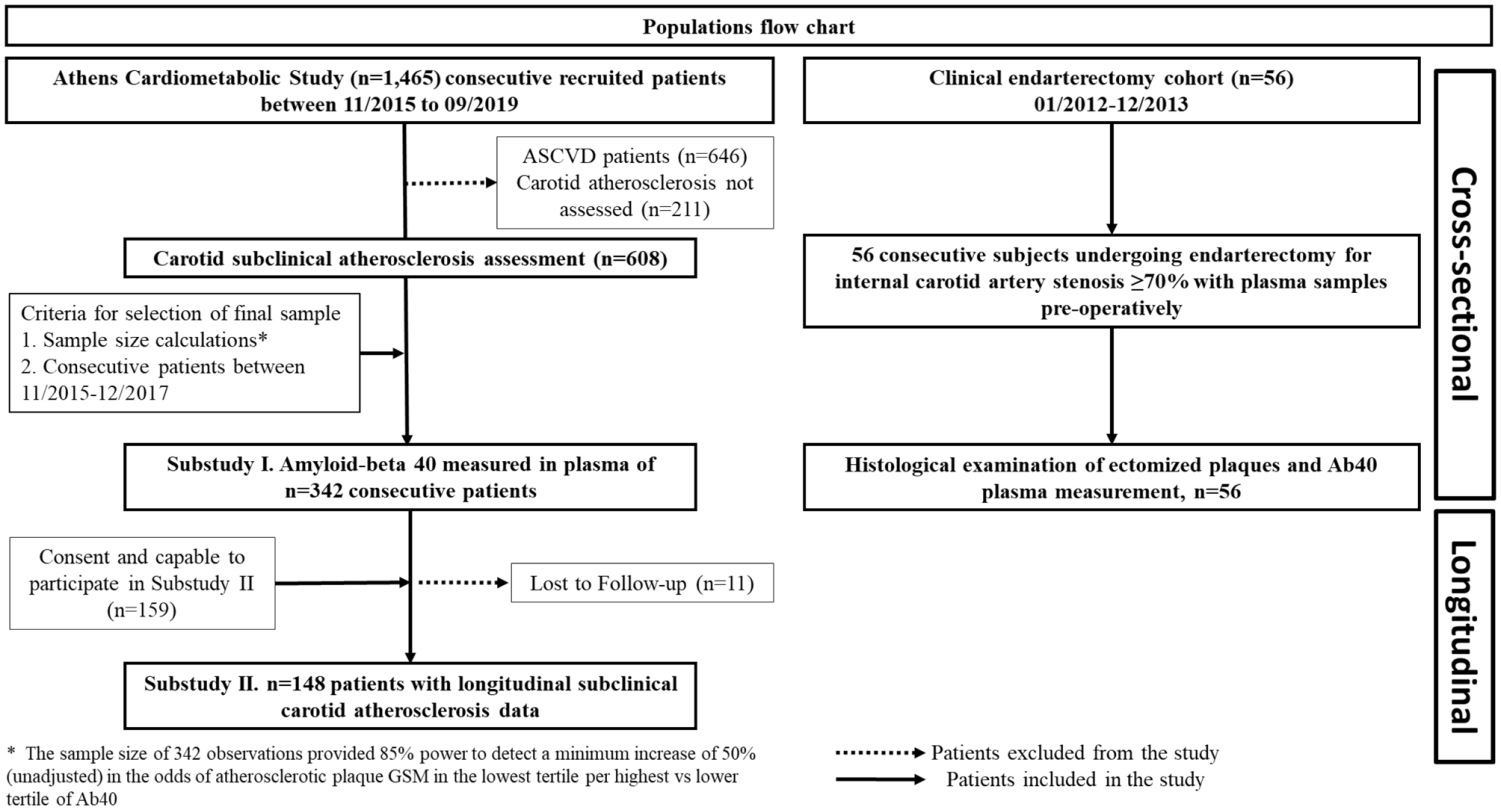
Flow Chart of the Study’s populations. ASCVD = atherosclerotic cardiovascular disease.

This is a registry comprised of two substudies: One cross-sectional including all consecutively recruited patients (Substudy I) and one longitudinal part including consecutive patients who were followed with yearly visits (Substudy II). This ongoing registry aims to stratify cardiovascular risk in participants being evaluated under primary or secondary ASCVD prevention settings. Recruitment is conducted at the Unit of Dyslipidemias and Atherosclerosis of the Department of Clinical Therapeutics, National and Kapodistrian University of Athens, as previously described^46^. Our study design included participants from both substudies, as follows:

*Substudy I.* We performed a retrospectively designed post-hoc analysis, for a pool of consecutively recruited participants between 2015 and 2019 from the Athens Cardiometabolic Study. Participants were included based on the following eligibility criteria: 1. patients without clinically overt ASCVD disease and, 2. available carotid ultrasonography data at baseline^47^. These criteria resulted in a total population of *n*=608 patients. From this group, Ab40 was measured in plasma in a subgroup of *n*=342 consecutive patients consecutively recruited between 12/2015 and 12/2017, based on sample size calculations as described in the statistical methods (Fig. 4).

*Substudy II.* Regarding the longitudinal part, from the total *n*=342 patients included in Substudy I, participants who consented and could attend successive visits for carotid atherosclerosis progression re-assessment, were included (*n*=159). Among them, *n*=11 were lost to follow-up and *n*=148 were included for analysis (Fig. 4).

All participants provided written consent for participation in the registry. The current study was conducted according to the principles of the Declaration of Helsinki and the Local Ethics Committee of Alexandra General Hospital approved the study’s protocol (13/26.11.2015).

#### Endarterectomy severe carotid stenosis cohort

Between January 2012 and December 2013, carotid plaques were prospectively collected from *n* = 56 consecutive patients, with internal carotid artery stenosis ≥ 70% who underwent carotid endarterectomy, as previously described^48^. Demographic and clinical data along with medication, and comorbidities were recorded (Table 4). Neurological evaluation of all patients was performed preoperatively to classify them as symptomatic based on the following: presence of stroke, brain infarcts, transient ischemic attacks and amaurosis fugax or asymptomatic. Angiography of the carotid bifurcation was performed in all patients for this study. Severity of luminal stenosis was determined according to North American Symptomatic Carotid Endarterectomy Trial criteria^49^. The study was conducted in accordance with The Code of Ethics of the World Medical Association (Declaration of Helsinki). All subjects provided written consent for participation in the study and the protocol was approved by the Institutional Ethics Committee (Scientific and Ethic Committee of Hipokrateion University Hospital, PN1539).

Carotid plaque specimens were removed during the endarterectomy and one part was fixed immediately in 10% neutral-buffered solution with 4% formaldehyde for 24 h, and embedded in paraffin. Hematoxylin and eosin staining was performed for histological evaluation of the specimens. Atheromatous plaque morphology of each specimen was performed by two pathologists, blinded to the clinical data, using the American Heart Association classification of atherosclerotic plaques. Analysis was performed according to the presence of histologic features related to plaque vulnerability (i.e., intraplaque hemorrhage, ulcer, or thrombus) or with plaque stability i.e. calcification^50^. Plaques without high-risk features plaques were defined as calcified plaques without any features related to plaque vulnerability. Plasma Ab40 levels were associated with the characteristics of the plaques.

#### Carotid Ultrasonography—Carotid wall morphology characterisation

Carotid atherosclerosis was assessed in three paired segments of both left and right common carotid artery, carotid bifurcation and internal carotid artery scanned from three transducer angles using B-mode ultrasound imaging (14.0-MHz multifrequency linear array probe, Vivid 7 Pro, GE Healthcare, Chicago, Illinois, United States). Intima-media complex (IMC) was defined as the maximum distance between the leading edges of the lumen–intima and media–adventitia ultrasound interfaces at each carotid site, excluding atherosclerotic plaques, according to previous Guidelines^51^. IMC was measured on the far wall of all three paired segments, in the right and left common carotid artery (CC), carotid bifurcation (CB), and internal carotid (IC) artery from 3 transducer angles using B-mode ultrasound imaging. For CC, IMC was measured on the far wall of the CCA at least 5 mm below its end, along 10 mm length of a straight arterial segment in a plaque-free area. For CB and IC IMC, IMC was measured at the carotid bifurcation or ICA bulb in a region free of plaque, on a shorter length than 10 mm. In this study, IMC was only used as part of the sum of wall thickness, used as a marker of atherosclerotic burden, as defined below. Carotid plaques were defined as a clearly identified area of focally increased IMC greater than 1.5 mm or a focal structure encroaching into the carotid lumen 50% of the surrounding IMC value^51^. The ultrasonographic images were digitised and then imported into a semi-automated software system designed specifically for analysing plaque and IMC echogenicity^28–30^. Specifically, after normalising the gray levels GSM was determined by the software: 1. For IMC GSM, the semi-automatic edge detection system (AMS v3.3) was used in all three segments for IMC measurement and IMC GSM analysis and 2. Using the semi-automated software, we manually delineated the plaque, and the software determined the gray scale median and plaque area (Supplementary Fig. 3). Markers of interest included: 1. The minimum gray-scale median (GSM) of all carotid plaques, 2. The minimum GSM of all IMC sites, 3. The maximum plaque area, and 4. The total area of all carotid plaques. Sum of wall thickness was defined as the algebraic sum of maximum wall thickness from all 6 carotid sites, being either an atherosclerotic plaque or IMC^52–54^. The prognostic value of assessing the presence and extent of carotid atherosclerosis in non-stenotic plaques has been previously validated^52–54^ and is recognised as a ASCVD risk modifier by ESC Guidelines^26,27^. GSM of carotid plaques and IMC were implemented as a marker of plaque vulnerability, as previously described^31,55^. After a median follow-up of 35.5 months, carotid ultrasonography measurements were repeated in a subset of the population (*n* = 148) using the same protocol as in baseline.

### Laboratory variables and Biomarker Testing

#### Athens Cardiometabolic cohort

Fasting blood samples were acquired with venipuncture. Plasma and serum samples were stored at − 80 °C until procession. Concentrations of Αb40 in ethylenediaminetetraacetic acid (EDTA)-plasma samples were measured in the baseline samples using a reliable enzyme-linked immunosorbent assay (ELISA) kit manufactured by Biosource/Invitrogen in California, USA, as previously described^5–7^. The intra- and inter-assay coefficient of variance of the ELISA measurements in our Laboratory was reported to be less than 8%^5^ which is consistent with that reported in the literature^56^. The minimum detectable concentration of human Aβ40 was < 6 pg/ml (Biosource/Invitrogen, California, USA).

#### Endarterectomy severe carotid stenosis cohort

Ab40 levels were measured in plasma samples acquired via venipuncture pre-operatively and stored at − 80 °C until procession. Concentrations of Αb40 in EDTA-plasma samples were measured in the baseline samples using the same ELISA kit (Biosource/Invitrogen in California, USA) as that in the Athens Cardiometabolic cohort^5–7^.

### Statistical analysis

We applied multivariable logistic regression analysis to examine the independent association of carotid atherosclerosis markers (i.e., highest vs. lower tertiles for total and maximal plaque and lowest vs. higher tertiles for plaque GSM) with Ab40 levels (i.e., highest tertile vs lower tertile of Ab40). We implemented GSM tertiles given that the lowest tertile of GSM in our population was ≤ 34.3, which falls within the range of GSM values of 30–40, correlating with high-risk plaque features, as acknowledged by the American Society of Echocardiography Recommendations^57^ and as described in previous studies^20,21,33^. The multivariable model was adjusted for a set of biologically plausible confounders, including age, sex, hypertension, dyslipidemia, smoking status, diabetes mellitus andGFR (core model). Differences in vascular markers of interest between the two visits were examined with the Wilcoxon signed-rank test for continuous variables. We implemented multivariable logistic regression analysis to examine the association between changes in vascular markers (total and maximal plaque area and IMC and plaque GSM) and Ab40 levels after adjustment for the core model. We categorised the changes in vascular markers as follows: 1. increasing or persistently high tertiles of total and maximal plaque area vs. decreasing or persistently low tertiles of these markers and 2. decreasing or persistently low plaque GSM tertile vs. increasing or persistently high plaque GSM tertiles. Regarding power considerations, the sample size of 342 observations provided a 85% power to detect a minimum increase of 50% (unadjusted) in the odds of atherosclerotic plaque GSM in the lowest tertile per highest vs lower tertile of Ab40^58^. Type I error was predefined at 0.05 and the anticipated effect size was extrapolated from previously published data on determinants of GSM^45^. All statistical analyses were performed using SPSS 21 (IBM Corporation, Armonk, New York). Statistical significance was defined at *p* < 0.05 unless stated otherwise. All tests were two-tailed.

### Supplementary Information

Supplementary Information.

## Data Availability

Data are available upon reasonable request to Prof. Kimon Stamatelopoulos.
