# Supplementary material for: Amyloid beta is associated with carotid wall echolucency and atherosclerotic plaque composition
Source: Sci Rep. 2024 Jun 28;14:14944. doi: 10.1038/s41598-024-64906-8 (PMC11213915; doi:10.1038/s41598-024-64906-8)
Supplement: Supplementary file 1 — Supplementary Information. [file 41598_2024_64906_MOESM1_ESM.docx]

**Supplementary Material**

Dimitrios Delialis, MD^1*^, Georgios Georgiopoulos, PhD^1*^, Simon Tual-Chalot, PhD^2^, Lasthenis Angelidakis, MD^1^, Evmorfia Aivalioti, MD^1^, Georgios Mavraganis, MD^1^, Kateryna Sopova^3,4^, Antonios Argyris, PhD^1^, Peggy Kostakou, PhD^1^, Christina Konstantaki, MD^1^, Maria Papaioannou, MD^1^, Diamantis Tsilimigras, MD^5^, Konstantinos Chatoupis, MD^1^, Achilleas A. Zacharoulis, MD^6^, George Galyfos, PhD^5^, Fragiska Sigala, PhD^5**^, Konstantinos Stellos, MD^2,3,7,8**^, Kimon Stamatelopoulos, PhD^1,2**^

^1^Department of Clinical Therapeutics, National and Kapodistrian University of Athens Medical School, Athens, Greece.

^2^Biosciences Institute, Vascular Biology and Medicine Theme, Faculty of Medical Sciences, Newcastle University, Newcastle Upon Tyne, UK.

^3^Department of Cardiovascular Research, European Center for Angioscience (ECAS), Heidelberg University, Mannheim, Germany.

Department of Cardiology, University Hospital Mannheim, Mannheim, Germany

^5^First Department of Propaedeutic Surgery, Hippocrateion Hospital, Medical School, University of Athens, Athens, Greece.

^6^Second Cardiology Department, Evangelismos Hospital, Athens, Greece

^7^German Centre for Cardiovascular Research (DZHK), Partner Site Heidelberg/Mannheim, Germany.

^8^Department of Cardiology, Angiology, Haemostaseology and Medical Intensive Care, University Medical Centre Mannheim, Medical Faculty Mannheim, Heidelberg University, Mannheim, Germany.

*First Equal Authorship

** Senior Equal Authorship

**Address for Correspondence:** Kimon Stamatelopoulos, MD, Vascular Laboratory, Department of Clinical Therapeutics, Alexandra Hospital, Medical School, National and Kapodistrian University of Athens, PO Box 11528, 80 Vas. Sofias Str., Athens, Greece. Email: kstamatel@med.uoa.gr

or

Konstantinos Stellos, MD, Department of Cardiovascular Research, European Center for Angioscience, Heidelberg University, Ludolf-Krehl-Straße 13-17, D-68167 Mannheim, Germany. Email: konstantinos.stellos@medma.uni-heidelberg.de

**Methods**

*Population*

History of CAD included: 1. history of hospitalization for acute myocardial infarction at time of screening, both ST-segment elevation myocardial infarction and NSTE-ACS, or 2. stable CAD defined as one of the following: a. Patients with history of stable angina or acute coronary syndrome ≥ 6 months, b. Patients who underwent percutaneous coronary intervention or coronary artery bypass graft ≥ 6 months and c. Patients with cardiac imaging indicative of CAD (coronary atheromatous plaque causing ≥ 50% lumen stenosis), stress echocardiography positive for ischemia, myocardial perfusion single-photon emission computed tomography detecting ischemia or treadmill test positive for ischemia.

Exclusion criteria included: history of clinically overt ASCVD or documented with imaging (CAD, ischemic stroke or transient ischemic attack and peripheral artery disease), withdrawal of consent, life expectancy <1-year, severe valvular heart disease, end-stage heart failure (New York Heart Association functional assessment) IV or ejection fraction <30%), already scheduled coronary revascularization at baseline visit, end-stage renal failure, active malignancy, autoimmune and infectious diseases. Medical history was obtained from all patients at baseline visit.

*Laboratory variables and Biomarker Testing*

Fasting blood samples were collected for standard biochemical lipid profile, including total cholesterol, triglycerides, low-density lipoprotein cholesterol (LDL-C) and high-density lipoprotein cholesterol (HDL-C) and high-sensitivity C-reactive protein (hs-CRP). LDL-C was measured using the Friedewald equation^1^.

**Statistical analysis**

Baseline characteristics were summarized using means and percentages for continuous and categorical variables, respectively. The Independent t-test and Mann-Whitney test were used to compare patients’ characteristics in the lower vs. highest tertiles of Ab40 for continuous variables; the chi-squared test was employed for categorical variables.

| **Supplementary Table 1. Association of the interaction between sex and Ab40 levels on carotid atherosclerosis indices (N=342)** | |
| --- | --- |
|  | Cross-sectional analysis |
| OR (95% CI) | |
| Maximal plaque area (highest tertile) | 1.25 (0.64 / 2.49)  P=0.484 |
| Total plaque area (highest tertile) | 1.50 (0.74/ 3.09)  P=0.241 |
| IMC GSM  (lowest tertile) | 1.55 (0.77 / 3.09)  P=0.217 |
| Plaque GSM  (lowest tertile) | 1.55 (0.72 / 3.32)  P=0.259 |
| OR represents the odds ratio for a patient with higher Ab40 levels (1-SD increase) to have carotid atherosclerotic indices (total and max plaque area) in the highest tertile and GSM in the lowest tertile.  Multivariable model includes sex, age, smoking status, history of hypertension, dyslipidemia, history of diabetes mellitus, and glomerular filtration rate.  IMC: intima-media complex, GSM: grey-scale median | |

| **Supplementary Table 2. Comparison of characteristics of patients without available follow-up (n=194) and with available follow-up (n=148) measurements of vascular indices** | | | |
| --- | --- | --- | --- |
|  | Patients without follow-up (n=194) | Patients with available follow-up (n=148) | p-value |
| Sex (male) | 80 (41.2) | 60 (40.5) | 0.768 |
| Age (years) | 58.1 (14.5) | 56.7 (8.8) | 0.377 |
| Smoking, % | 62 (33.5) | 42 (28.7) | 0.671 |
| Hypertension, % | 75 (38.7) | 52 (35.1) | 0.314 |
| Hyperlipidemia, % | 98 (50.5) | 66 (44.5) | 0.264 |
| Hypolipidemic treatment, % | 49 (25.3) | 35 (23.6) | 0.454 |
| Diabetes mellitus, % | 29 (14.9) | 13 (8.8) | 0.153 |
| BMI (kg/m^2^) | 27.7 (4.5) | 27.3 (5.0) | 0.481 |
| GFR (ml/m^2^/min) | 113.7 (43.9) | 121.9 (38.6) | 0.120 |
| LDL-C (mg/dl) | 128.0 (42.8) | 135.2 (36.7) | 0.142 |
| SBP (mmHg) | 126.7 (18.3) | 129.3 (19.7) | 0.249 |
| DBP (mmHg) | 73.4 (10.9) | 72.0 (10.8) | 0.283 |
| P-values are derived from independent Student’s t-test for continuous variables and the Pearson’s chi squared test for categorical variables. BMI: Body mass index, GFR: Glomerular filtration rate, LDL-C: low-density lipoprotein cholesterol, HDL-C: high-density lipoprotein cholesterol, SBP: Systolic blood pressure, DBP: Diastolic blood pressure. | | | |

| **Supplementary Table 3. Comparison of vascular indices between baseline and follow-up** | | | | |
| --- | --- | --- | --- | --- |
|  | Baseline (Ν=342) | Follow-up (Ν=148) |  | p-value |
| Maximum plaque area, [mm^2^], median (IQR) | 24.8 (15.8, 41.9) | 37.1 (24.1, 63.94) |  | 0.007 |
| Total plaque area, [mm^2^], median (IQR) | 34.4 (18.0, 61.9) | 64.2 (31.5, 121.7) |  | 0.015 |
| IMC GSM, median (IQR) | 23.0 (15.0, 37.0) | 20.5 (16.5, 26.0) |  | 0.633 |
| Plaque GSM, median (IQR) | 43.0 (31.0, 55.0) | 36.0 (28.0, 50.5) |  | 0.936 |
| In patients with increasing IMC GSM n=78 / plaque GSM n=25 | | | | |
| IMC GSM, median (IQR) | 34.0 (25.0, 43.0) | 27.0 (23.0, 21.0) |  | 0.186 |
| Plaque GSM, median (IQR) | 50.5 (42.7, 61.0) | 47.1 (32.7-67.7) |  | 0.292 |
| In patients with decreasing IMC GSM n=70 / plaque GSM n=23 | | | | |
| IMC GSM, median (IQR) | 19.0 (13.0, 31.0) | 15.0 (13.0, 16.5) |  | 0.083 |
| Plaque GSM, median (IQR) | 31.0 (27.0, 37.0) | 25.0 (14.5, 31.0) |  | 0.01 |
| IMC: intima-media complex, GSM: gray scale median. Continuous variables are presented as mean (SD). P-value derived from Wilcoxon Signed Rank Test. | | | | |

| **Supplementary Table 4. Association of the interaction between sex and Ab40 levels on the progression of carotid atherosclerosis indices (N=148)** | | | |
| --- | --- | --- | --- |
| Carotid atherosclerosis | | |  |
| Pattern of increasing or  persistently highest tertile of | | OR (95% CI) | |
| Maximal plaque area, [mm^2^], median (IQR) | | | 3.09 (0.62 / 15.55)  P=0.172 |
| Total plaque area, [mm^2^], median (IQR) | | | 3.21 (0.02 / 7.51)  P=0.193 |
| Pattern of decreasing or  persistently lowest tertile of |  | | |
| IMT GSM, median (IQR) | | | 1.29 (0.66 / 2.49)  P=0.441 |
| Plaque GSM, median (IQR) | | | 1.20 (0.86 / 1.73)  P=0.276 |
| OR represents the odds ratio for a patient with higher Ab40 levels (per 1-SD increase) to have increasing or persistently high carotid atherosclerotic indices (total and max plaque area) and decreasing or persistently low GSM (IMT and plaque) tertile.  Multivariable model includes sex, age, smoking status, history of hypertension, dyslipidemia, history of diabetes mellitus, and glomerular filtration rate.  IMC: intima-media complex, GSM: gray scale median | | | |

**Supplementary Figure 1**. Correlation matrix for Ab40 levels in the whole population (A, C) and in patients with GFR≥60ml/min/m^2^ (B, D) for plaque and IMC GSM. P-values are derived from Spearman’s rank correlation coefficient. Ab40: Amyloid-beta 1-40, GSM: grey scale median, IMC: intima-media complex.


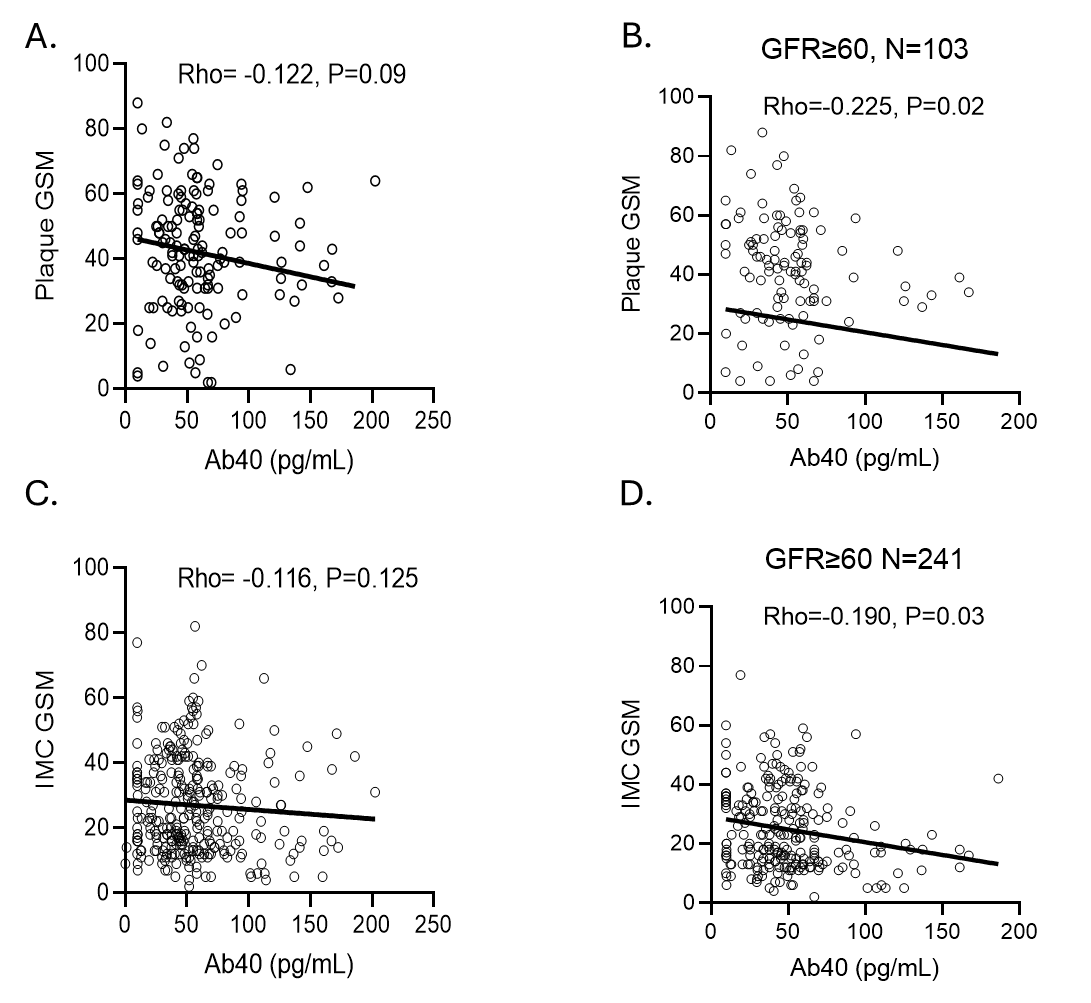


**Supplementary Figure 2.** Scatter plot for the correlation of Ab40 levels with: A. IMC and B. plaque GSM in the follow up. Changes in IMC (C, E) and changes in plaque GSM (D, F) in patients with persistently low / decreasing GSM levels and persistently high / increasing GSM levels. P-values are derived from Spearman’s rank correlation coefficient. Ab40: Amyloid -beta 1-40, GSM: grey scale median, IMC: intima-media complex, Delta: Difference between follow-up and baseline.


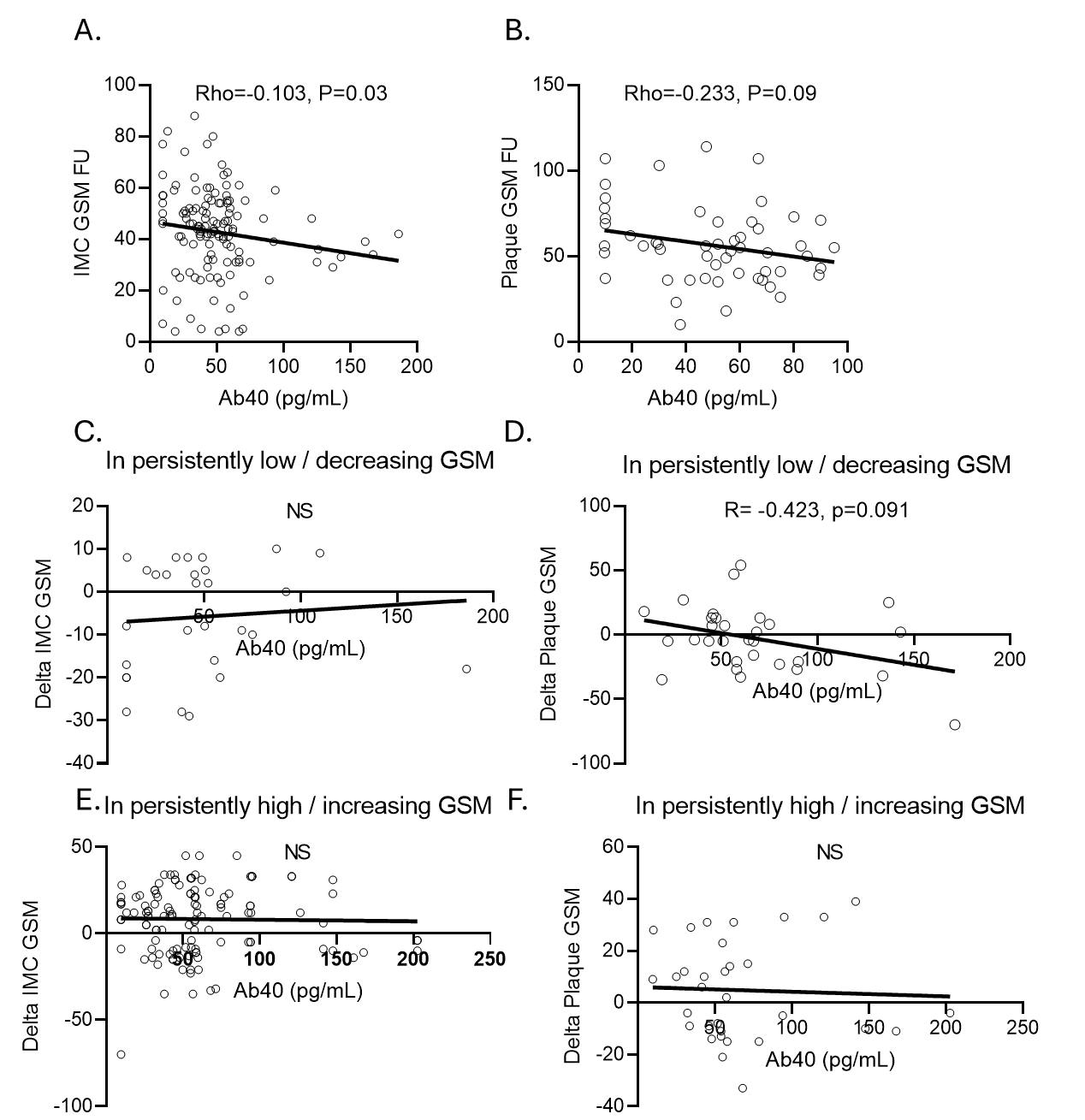


**Supplementary Figure 3**: Evaluation of the echogenicity of carotid wall using a dedicated software artery measurement system. A. Measurement of IMT GSM using a semi-automated method. B. Measurement of carotid plaque GSM after manually delineating the plaque, and the software determined the gray scale median and plaque area.


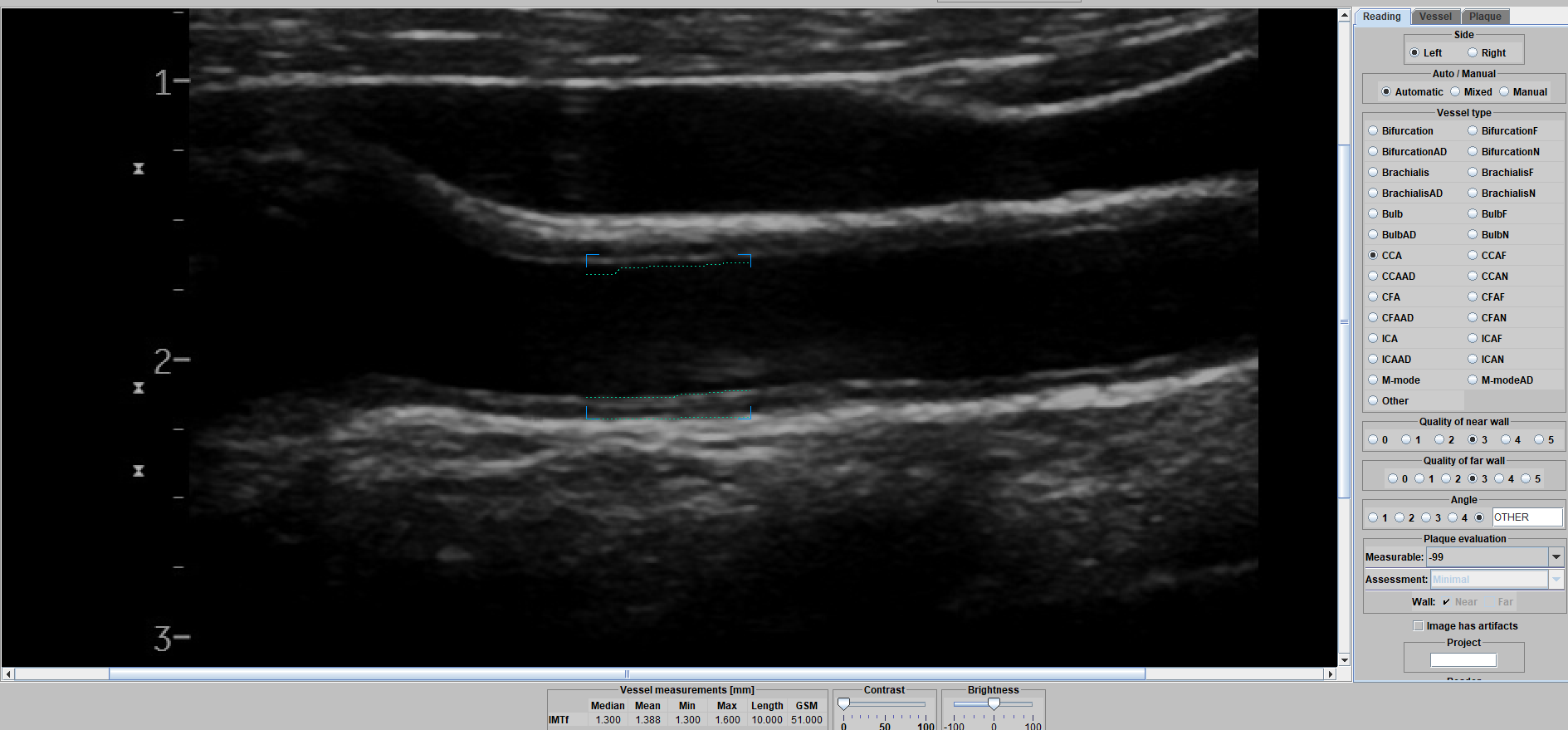


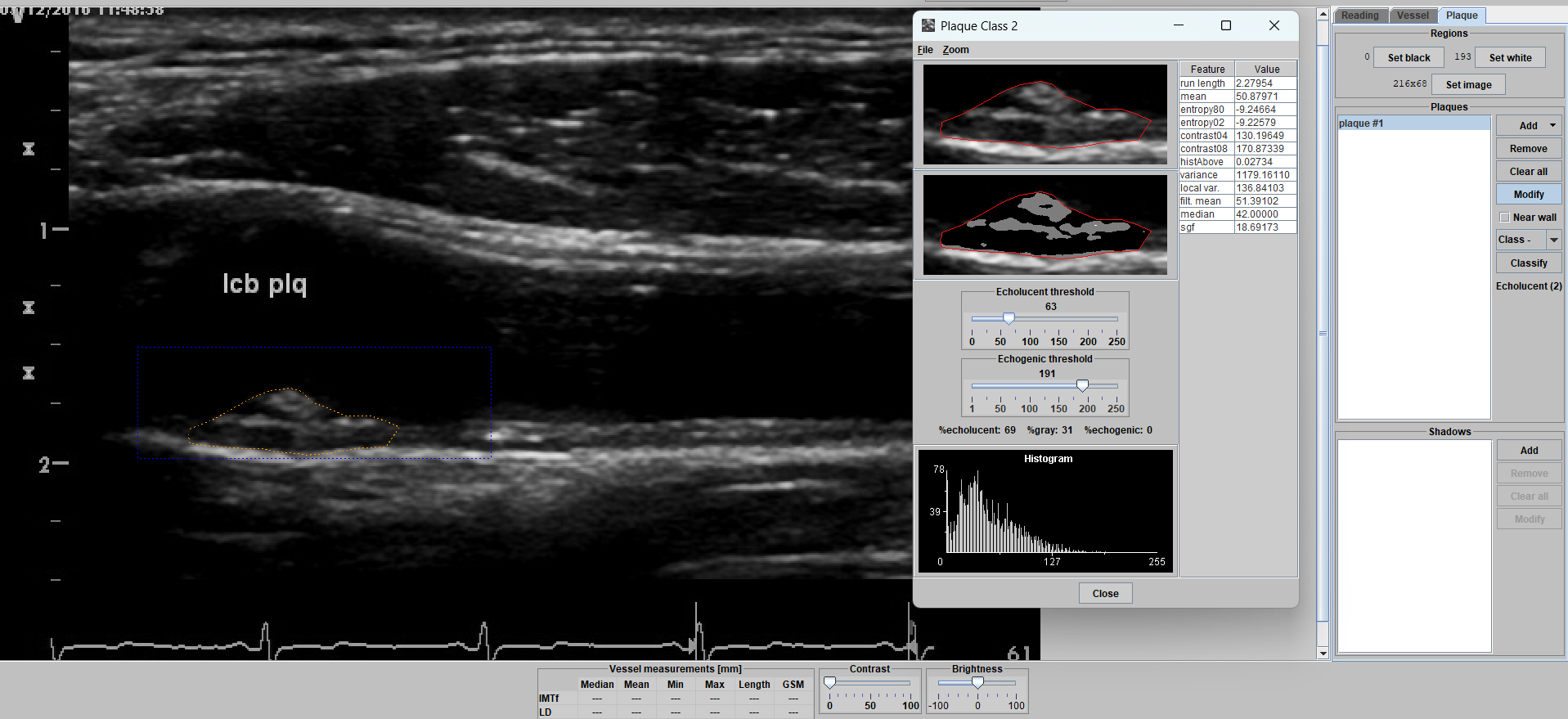


**References**

1 Stamatelopoulos, K. *et al.* Estimated pulse wave velocity improves risk stratification for all-cause mortality in patients with COVID-19. *Sci Rep* **11**, 20239 (2021). <https://doi.org:10.1038/s41598-021-99050-0>
